# Transcriptional Biomarkers for Treatment Monitoring of Pulmonary Drug-Resistant Tuberculosis: Protocol for a Prospective Observational Study in Indonesia

**DOI:** 10.3390/tropicalmed7110326

**Published:** 2022-10-22

**Authors:** Ida Parwati, Dian Ayu Eka Pitaloka, Lidya Chaidir

**Affiliations:** 1 Department of Clinical Pathology, Faculty of Medicine, Universitas Padjadjaran, Sumedang 45363, Indonesia; 2 Department of Pharmacology and Clinical Pharmacy, Faculty of Pharmacy, Universitas Padjadjaran, Sumedang 45363, Indonesia; 3 Center for Translational Biomarker Research, Universitas Padjadjaran, Sumedang 45363, Indonesia; 4 Department of Biomedical Sciences, Faculty of Medicine, Universitas Padjadjaran, Sumedang 45363, Indonesia

**Keywords:** tuberculosis, blood biomarker, gene expression, transcriptome, treatment response

## Abstract

Many blood-based gene expression biomarkers for monitoring tuberculosis (TB) treatment have been suggested so far, but promising biomarker results for drug-resistant TB treatment response have not been studied. This protocol presents a prospective observational study in Indonesia to profile the human blood transcriptome for predicting the response to drug-resistant TB treatment, focusing on pulmonary TB, and to adapt the specific RNA signature to the qRT-PCR platform. Longitudinal blood samples will be collected from 44 subjects with rifampicin resistant TB, confirmed by Xpert MTB/RIF, and 52 healthy controls. RNA-Seq will be performed to identify changes in the transcriptome following TB treatment. A discriminative RNA signature will be chosen and translated into a score for use in a quantitative PCR-based assay. This study will provide crucial information to guide the discovery and design of a clinically implementable tool to monitor the response of TB treatment.

## 1. Introduction

Indonesia is ranked as having the third highest tuberculosis (TB) burden worldwide, with approximately 570 thousand cases in 2019 [1]. The increasing prevalence of HIV and multi-drug resistance TB (MDR-TB, about 10,174 cases in 2019) poses serious challenges for effective control [1]. The diagnostic gold standard for active TB is finding *Mycobacterium tuberculosis* (MTB) in clinical specimens by culture or detecting MTB-specific nucleic acids by molecular methods [2,3]. However, culture methods need a complex infrastructure for proper safety and a long turnaround time, while molecular techniques are still costly. Sputum for the diagnosis is also problematic in many circumstances, for instance, production can be difficult (e.g., in children). The number of MTB bacteria in sputum can also vary widely, even from the same patient at the same time. Furthermore, the number of bacteria in sputum can be below detection in the early stages of infection and following treatment, resulting in people being either empirically treated for TB, receiving inappropriate preventive therapy, or not receiving TB therapy at all [4].

A further problem in the management of TB is the extended length of treatment. Poor treatment monitoring, and hence inadequate treatment, leads to worsening of a patient’s disease, increasing the potential for disease transmission and the risk of developing drug- resistant TB (DR-TB). To date, sputum culture conversion is the only biomarker of TB treatment monitoring [2]. However, it is time consuming and patients who have clinically improved may be unable to expectorate sputum and potentially incorrectly labelled as having a negative culture. Another marker, chest X-rays, is commonly used, but is generally slower than the clinical response and has low specificity in patients with previous lung damage [5]. Moreover, standardized interpretation of radiographic changes in response to treatment has not yet been established.

The World Health Organization (WHO) End TB Strategy calls for the development and implementation of TB non-sputum diagnostic tests [5], such as blood, for more comprehensive and straightforward diagnosis in the primary healthcare setting. RNA expression analysis has emerged as a powerful tool for predicting treatment response during therapy. It could reduce the treatment duration and costs or allow for early switching of anti-tuberculosis drugs when treatment is failing [6], and is also very useful for when good quality sputum sample cannot be obtained [7]. Several gene signatures have been reported in several studies and validated in various populations and target groups [8,9,10]. However, none of the studies explored the RNA signatures to monitor treatment in drug-resistant patients.

Therefore, the overarching aim of this study is to explore blood transcriptomic signatures in Indonesian pulmonary TB (PTB) patients with applicability for predicting treatment response in drug-resistant TB treatment. We will undertake a genomic approach to discover a genetic signature for DR-TB and integrate this with previous reported signatures. This will be combined with clinical and public health expertise to interpret what information is most useful and at what stage of the treatment process a test would provide the most benefit. The most specific expression pattern of the signature genes associated with changes during therapy will be selected and will be adapted to quantitative real-time polymerase chain reaction (qRT-PCR). A TB score will be developed based on the gene-level summarized intensities using raw Ct values generated from the qRT-PCR.

## 2. Design/Methods

### 2.1. Study Design

The study will be a cohort in which patients will be consecutively recruited in the MDR-TB clinic of Dr. Hasan Sadikin Hospital (RSHS), Bandung, Indonesia. RSHS is a tertiary hospital in West Java province that identifies up to 250 new cases of TB with up to 150 Xpert/RIF-resistant annually. All patients with suggestive for PTB (cough > 2 weeks) will be tested with Xpert MTB/RIF assay. The study was approved by the Institute Review Board of the Faculty of Medicine, Universitas Padjadjaran (037/UN6.KEP/EC/2021) in March 2021. Written informed consent will be obtained from all participants.

### 2.2. Eligibility Criteria

**Figure 1:**
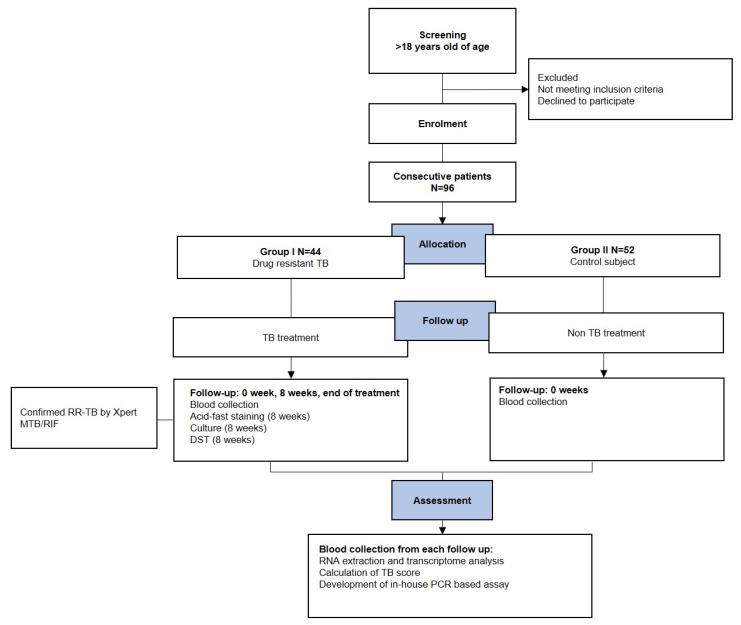
Research flow of the study. The RSHS-UNPAD manages anti-TB treatment for all enrolled participants according to the WHO recommendation. Blood sampling will be collected from the TB clinic, RSHS Bandung. TB, tuberculosis; RR-TB, Rifampicin-resistant tuberculosis; DST, drug susceptibility test; PCR, Polymerase chain reaction.

The study population includes 1000 presumptive TB, both new and re-treatment adult PTB cases who visit the TB clinic RSHS Bandung. Only rifampicin-resistant (RR) PTB patients will be followed up in this study, regardless of their HIV status. Group I will consist of active PTB cases confirmed as RR-PTB by Xpert MTB/RIF testing (Table 1). Further phenotypic drug susceptibility testing (pDST) by MGIT960 will be performed for rifampicin and other first and second line drugs following guidelines from the National TB Program. Group II will be the control group, consisting of healthcare workers from the TB hospital suspected of not having TB, proven by chest X-ray mycobacterial examinations, and latent TB infection using interferon-gamma release assay (Quantiferon TB-Gold assay). Control subjects with signs, symptoms, and chest X-ray radiograph (CXR) results suggestive of active TB, a history of prior anti-TB treatment, and a positive IGRA test will be excluded. HIV status is not tested in the control subjects (Figure 1).

### 2.3. Planned Sample Size

The sample is calculated by the diagnostic test formula for the receiver operating characteristic (ROC) [11]. Based on the calculation, the minimum sample size in this study is 48 per group. The prevalence of TB response treatment in drug-resistant TB is estimated at 46%. So, the total sample is 96 people, divided by 44 subjects for drug-resistant TB and 52 subjects for healthy control.

### 2.4. Planned Study Period

The study will be carried out for three years (2021–2024) and the work plan can be summarized as follows:

First year:

Ethics approval has been issued. We have initiated the recruitment of patients and treatment follow-up in June 2021, and initiation of the RNA-seq work.

Second year:

We will continue patient recruitment and data collection. RNA extraction will be conducted, continued to RNA sequencing. A first comparison will be made between transcriptomic assay versus standard diagnostics work.

Third year:

The end of clinical and sample collection. We will assess the relative performance of different risk scores for determining treatment success in drug-resistant TB and develop an in-house PCR-based assay.

#### 2.4.1. Specimen Collection and Processing

For transcriptomic analysis, 2.5 mL of blood samples at three-time points of the disease spectrum (baseline: 0 weeks, treatment phase: 8 weeks, and convalescent phase: end of treatment) will be obtained and collected in PAXgene Blood RNA tubes (Qiagen, Düsseldorf, Germany). The sample will be gently inverted and stored at −80 °C within two hours of collection. For mycobacteriology examinations, sputum sample will be tested with Xpert MTB/RIF at baseline. Sputum from patients with RIF-R will be cultured and processed for pDST using MGIT960 according to the standard guideline. MTB bacteria isolated from the positive culture in MGIT (Becton Dickinson, Franklin Lakes, NJ, USA) tube will be cryopreserved in liquid Middlebrook 7H9 (Burlington, NY, USA) medium containing 20% glycerol and stored in duplicate at −80 °C.

#### 2.4.2. Anti-TB Treatment

The RSHS-UNPAD manages anti-TB treatment for all enrolled participants according to the WHO recommendations. RSHS-UNPAD is the teaching hospital for the Faculty of Medicine Universitas Padjadjaran and a tertiary reference hospital for West Java. A short treatment regimen for rifampicin-resistant TB (RR-TB) is recommended for those with new cases of rifampicin resistance or treatment failure. The RR-TB regimen should include at least 7 oral anti-TB drug combinations for 9–12 months [12]. Sputum smears and culture will be analyzed monthly until remaining negative. We define drug-resistant TB treatment outcomes by the WHO definition (Table 2) [13].

#### 2.4.3. RNA Extraction and Integrity Assessment

RNA is extracted from whole blood using the PAXgene™ Blood RNA System Kit (Qiagen, Düsseldorf, Germany) employing an amended version of the manufacturer’s guidelines. Briefly, the samples are removed from −80 °C and incubated at room temperature for 2 h to ensure complete lysis. Following lysis, the tubes are centrifuged for 10 min at 5000× *g* (Boseo M-24 centrifuge, Hamburg, Germany), the supernatant is decanted, and 500 μL of RNase-free water is added to the pellet. The tube is vortexed to thoroughly resuspend the pellet, centrifuged for 10 min at 5000× *g*, and the entire supernatant discarded. The remaining pelleted lysate is re-suspended in 360 μL of buffer BR1 by vortexing, and the manufacturer’s protocol is followed from this step [14,15].

Freshly extracted RNA is measured using a NanoDrop ND-1000 UV-visible spectrophotometer (Thermo Fisher, Waltham, MA, USA). The software displays the concentration in ng/μL. There is also a quality output, which provides 260/280 and 260/230 ratios enabling purity estimations. RNA integrity is additionally assessed using the Agilent 2100 Bioanalyzer (Agilent Technologies, Santa Clara, CA, USA), and poor quality samples are excluded from the analysis [15].

#### 2.4.4. Transcriptome Sequencing and Bioinformatic Analysis

Approximately 500 ng of RNA from each sample is used to prepare individually barcoded strand-specific RNA-seq libraries. Libraries will be constructed using ScriptSeq™ Complete Kit (Illumina). Next-generation sequencing (NGS) will be performed using an Illumina HiSeq 2500 with one 150-bp end run at the National Genome Center, Eijkman Institute for Molecular Biology, Jakarta, Indonesia. Reads will be aligned to human sequences using STAR [16]. The differential expression will be analyzed in SeqMonk v.1.48.1 [17] after quantifying raw counts, strand-specific, merged isoforms, using the R (v3.2.2) package DESeq2 [18]. Cuffdiff v.2.2.1.2 [19] is used for differential expression analysis. The differential expression genes between two samples are selected based on the fold change (greater than 2) and the false discovery rate (less than 0.05). To understand the functions of the differentially expressed genes, gene ontology functional enrichment and Kyoto encyclopedia of genes and genomes pathway analysis will be carried out by Goatools v.0.8.2 [20], and KOBAS v.3.0 [21]; differentially expressed genes are significantly enriched in the gene ontology terms and metabolic pathways when their Bonferroni-corrected *p*-value is less than 0.05.

The expression pattern of differentially expressed genes identified by RNA-Seq is validated using Real-Time PCR. The total RNA is reverse-transcribed into cDNA, and the gene transcription levels are analyzed using a SYBR Green PCR kit (Toyobo, Osaka, Japan) on Light Cycler 96 Detector system (Roche, Basel, Switzerland). Primer sequences of the selected genes are designed using Primer 3 v.2.6.0 [22]. The relative gene transcription levels are calculated using the 2−ΔΔCT method; beta-actin is used as the reference gene, and its transcription is constant among exposure groups. The Pearson’s correlation of log10 (fold-change) between qPCR and RNA-Seq is 0.80, indicating the accuracy and reliability of the RNA-Seq based transcriptome analysis [23].

#### 2.4.5. Development of In-House PCR Based Assay

We will develop an inexpensive, rapid, and specific method based on TaqMan real-time PCR to generate a large panel of genes for evaluating the monitoring therapy based on the signature genes. The development steps include (1) primer design and probes; (2) cDNA synthesis; (3) TaqMan real-time PCR; (4) specificity panel; (5) sensitivity and limit of detection; and (6) inter-and intra-assay to evaluate precision. Primers and TaqMan probe are designed using the AlleleID7 v.7.71 [24] and synthesized by Macrogen Company (Korea). The RNA is reverse-transcribed into cDNA and analyzed using a real-time PCR Master Mix (Thermo Fisher) and TaqMan probe. Twenty human genomes are obtained to evaluate the specificity of the assay. The first step of specificity assessment is performed using BLASTn analysis. The sensitivity and limit of detection of the target in the samples are achieved by diluting standard signature genes with 6, 12, 25, 50, and 100 copies/μL and analyzed by real-time PCR. Moreover, 10-fold serial dilutions from 10_2_ to 10_7_ copies/μL of the standard signature genes are prepared to evaluate the intra- and inter-assay, respectively. Each one is tested in triplicate using real-time PCR.

#### 2.4.6. Calculation of TB Score

We will next calculate the TB score computed from the gene-level summarized intensities. The TB score can be calculated using qRT-PCR data where raw Ct values of the genes are used [8]. In this proposed study, we will take advantage of frequent longitudinal sampling patients to plot their qRT-PCR-based risk score over time. qRT-PCR gene expression data is quality controlled using scripts generated in R, and signature scores are calculated. The difference in raw Ct is computed for each transcript pair, producing the log-transformed ratio of expression and the measured ratio of proportions in a look-up table for the given pair of transcripts. The corresponding score will be assigned, then assigned in the look-up table to the ratio. If the measured ratio is more significant than all ratios in the relevant column of the look-up table, we will give a score of 1 to the ratio. The average over the scores is computed generated from the set of pairs. The average overall ratios are calculated if any assays fail on the sample, not including the failed assays. The resulting average is the final score. All TB scores, except for those in the discovery cohort, are generated by blinded laboratory personnel. Receiver operating characteristic (ROC) AUCs are developed and compared using the pROC [25] and verification [26] packages in R. 

### 2.5. Outcome Measures/End Points

Our primary objective is to use a cohort analysis of blood samples from drug-resistant TB patients longitudinally sampled to derive a predictive TB score to evaluate TB treatment response. Other key objectives to be addressed in this prospective observational study are as follows: (1) to discover the biomarker gene signature in Indonesian TB patients using RNA-Seq (2) to develop a TB score and PCR-based in-house assay using targets specific transcriptome profile.

## 3. Discussion

RNA expression analysis has emerged as a powerful tool for understanding disease biology, including infectious diseases [27]. A blood sample is an alternative for patients, especially from high-quality sputum samples, which cannot be obtained primarily in TB cases. Transcriptional profiling data on peripheral blood samples from TB patients can distinguish between the different stages of TB infection and other conditions [28,29,30]. In this study, we aim to enrich the exploration of already reported biomarkers with drug resistance TB which is still unavailable.

Anderson et al. [29] performed prospective cohorts to evaluate the transcriptional signatures of host blood to distinguish TB from other diseases. They identified a 51-transcript signature and validated it in various populations and target groups such as HIV or coinfection with other diseases, children, and household contacts/latently infected individuals [29]. These results could have many prospective clinical implications, such as predicting TB disease progression, treatment efficacies, and clinical outcomes.

Another study reported a transcriptional signature in specific-antituberculosis (anti-TB) treatment response from blood, comparing untreated active TB patients, during treatment, and after anti-TB treatment. The study found 664-transcript signatures related to active TB and 320-transcript signatures related to anti-TB treatment significantly diminished after 2 weeks of treatment and continued to diminish until the end of treatment [9]. Its finding suggested the transcriptomic’s potential as early surrogate biomarkers of successful treatment response.

Kwan et al. [31] had conducted a study by undertaking a novel anti-TB therapy trial in 24 patients, where 4 blood samples were drawn over the 24 weeks of standard TB therapy to allow RNA sequencing of the blood transcriptome. They identified a 16-gene RNA response signature composed of previously identified genes from other studies, as those genes recognize active TB disease [31]. Combining these gene expression levels successfully created an active TB disease “risk score”, as previously described in their work [28]. They were also able to correlate the quantitative PCR results, further measuring relative amounts of gene transcripts. The RNA sequence data measured relative transcriptome counts from the whole blood patient and measured the performance of reducing the PCR assays in diagnosing active TB [32].

More specifically for treatment monitoring, several sets of RNA signatures have been well explored and validated in various cohorts, namely Sweeney3 [33], RESPONSE5 [33] and RISK6 [8]. All those signatures were successfully adapted to and confirmed on the qRT-PCR platform, which respectively measured the relative number of gene transcripts (TB score). Those assays composing the signatures were then pooled and mined to systematically identify small gene subsets that were able to capture differential responses to TB treatment.

Although several host response-based transcriptional signatures have been described for monitoring TB treatment, profiling of these signatures is subjected to potential bias, including heterogenicity in MTB strains, confounding by age or coinfection with HIV, and the presence of other lung diseases [9,29]. However, individual reports showed that the performance of different sets is not affected by age, HIV coinfection, and prior BCG vaccination [8,32,33]. Importantly, validation of different sets using clinical samples from multiple countries demonstrates their robustness to differences in the genetic background of patients and TB strains [32,33].

Since drug-resistant TB may have slower sputum conversion to negative versus drug-sensitive TB, we will enrich the study population with longitudinal samples collected from patients with confirmed RR-TB as a surrogate for MDR-TB, as the RNA signatures associated with TB treatment monitoring in DR-TB are poorly explored. Due to extended treatment of DR-TB, we cannot avoid the possibility of losing follow-up patients when culture results take 40 to 60 days to declare negativity. This could be a challenge for our study. Possible limitations include the unavailability of positron emission tomography and computerized tomography (PET-CT) imaging, so we cannot correlate RNA signature scores developed in this study with metabolic activity in lung lesions as measured by total glycolytic activity index (TGAI) from PET-CT [8]. We also are not performing the validation study in an independent cohort. Intensive follow-up from the research team and strictly performing other biometrics should hopefully reduce the possible challenges and limitations.

## 4. Conclusions

This prospective study is designed to establish if early changes in blood transcriptional responses can be observed during DR-TB treatment. We believe that this study of blood-based gene expression biomarkers is urgently needed for a comprehensive and straightforward diagnosis in the primary healthcare setting, allowing for rapid anti-TB treatment and monitoring. Adoption of the qRT-PCR score as the main outcome of this study will provide a convenient platform for stratifying patients according to their risk of treatment failure. All the results will provide crucial information to guide the discovery and design of possible active case-finding interventions to enhance TB control in Indonesia. However, further validation in diverse and independent cohorts that represent real-world TB patients needs to be conducted.

## Figures and Tables

**Table 1:** Inclusion and exclusion criteria.

Inclusion and Exclusion Criteria	
Inclusion Criteria	Adult pulmonary TB patients (>18 years old) who visit TB clinic RSHS Bandung
	Newly diagnosed as rifampicin-resistant TB (RR-TB) by Xpert MTB/RIF, regardless of HIV status
Exclusion Criteria	Clinical TB patients without proven by laboratory examinations
	Patients diagnosed with extrapulmonary TB
	Patients who plan to start their treatment in another clinic during the period of research
	Hepatitis patients with interferon treatment, which is known to aggravate TB infection
	Patients diagnosed with a malignancy
	Patients who are undergoing chemotherapy and using immunomodulators
	Patients who have started TB treatment in the past week
	Patients who are using oral steroids for more than two weeks

**Table 2:** Case definitions used in the study.

Type of Case	Definition
Presumptive TB	A patient who presents with symptoms or signs suggestive of TB and previously known as a TB suspect
Pulmonary TB	Any bacteriologically confirmed or clinically diagnosed case of TB involving the lung parenchyma or the tracheobronchial tree
Extrapulmonary TB	Any bacteriologically confirmed or clinically diagnosed case of TB involving organs other than the lungs, e.g., pleura, lymph nodes, abdomen, genitourinary tract, skin, joints and bones, meninges
Relapse patients	Patients who have previously been treated for TB, were declared cured or treatment completed at the end of their most recent course of treatment, and are now diagnosed with a recurrent episode of TB (either a true relapse or a new episode of TB caused by reinfection)
Cured TB	Treatment completed as recommended by the national policy without evidence of failure and three or more consecutive cultures taken at least 30 days apart are negative after the intensive phase
Treatment completed	Treatment completed as recommended by the national policy without evidence of failure BUT no record that three or more consecutive cultures taken at least 30 days apart are negative after the intensive phase
Treatment failed	Treatment terminated or need for permanent regimen change of at least two anti-TB drugs because of: — Lack of conversion by the end of the intensive phase, or — Bacteriological reversion in the continuation phase after conversion to negative, or — Evidence of additional acquired resistance to fluoroquinolones or second-line injectable drugs, or — Adverse drug reactions (ADRs)
Died	A patient who dies for any reason during the course of treatment
Lost to follow-up	A patient whose treatment was interrupted for 2 consecutive months or more
Not evaluated	A patient for whom no treatment outcome is assigned. This includes cases “transferred out” to another treatment unit and whose treatment outcome is unknown
Treatment success	The sum of cured and treatment completed

## Data Availability

Not applicable.
